# Early changes in the CD8 T Cell immunodominance hierarchy in primary HIV infection prior to seroconversion

**DOI:** 10.1186/1742-4690-9-S2-P244

**Published:** 2012-09-13

**Authors:** N Keane, C Almeida, A Chopra, D Cooper, E Demaine, S Mallal, M John

**Affiliations:** 1 Murdoch University, Perth, Australia

## Background

Identification of the earliest CD8 T-cell responses against HIV may help select critical viral targets for inclusion in an HIV vaccine. We describe changes to the earliest detected CD8 T-cell responses and changes in genetic sequence encoding targeted epitopes in an individual who presented with Fiebig stage II, clade C acute HIV-infection, 27 days after sexual transmission.

## Methods

We examined HLA-restricted CD8 T-cell responses by IFNγ ELISpot and HIV Gag, Pol, Nef and Env sequence by 454 deep sequencing over 6 timepoints, from days 27-118 after HIV-transmission. HIV-specific IFNγ responses were detected against ten HIV epitopes in Gag, Nef and Env at day 27 post HIV-transmission when the patient’s CD4 T-cell count was at a nadir of 224 cells/μl, the plasma HIV RNA >106 copies/ml and prior to any detectable p24 antibody.

## Results

Immunodominant responses were detected to the HLA-B*07:02 restricted Env IIRRIRQGL (IL9) epitope and the B*07:02 Gag GPGHKARVL (GL9) epitope (>4000SFU). A detectable but weaker response was observed to HLA-B*08 Nef FLKEKGGL (FL8) epitope (1010SFU). These responses declined over subsequent timepoints to 470 and 1630SFU on day 118 coincident with a 2 log fall in the plasma viral load, a rise in the CD4 T cell count to 533 cells/µL and antibody seroconversion. The Nef FL8 became the immunodominant response after day 34. IFNγ responses broadened from 10 responses at day 27 to 23 responses by day 118. Analysis of Gag, Nef and Pol genes by 454 deep sequencing showed no evidence of escape within the targeted epitopes as a cause of their decline over time.

## Conclusion

Early changes in the CD8 T-cell immunodominance hierarchy are apparent in acute HIV-infection prior to seroconversion, including early immunodominant targeting of Env epitopes. Subsequent broadening of the CD8 T-cell response was not associated with CD8 T-cell escape in this case.

